# Cell Type-Specific Arousal-Dependent Modulation of Thalamic Activity in the Lateral Geniculate Nucleus

**DOI:** 10.1093/texcom/tgab020

**Published:** 2021-03-16

**Authors:** Benedek Molnár, Péter Sere, Sándor Bordé, Krisztián Koós, Nikolett Zsigri, Péter Horváth, Magor L Lőrincz

**Affiliations:** Department of Physiology, Anatomy and Neuroscience, Faculty of Sciences, University of Szeged, 6726 Szeged, Hungary; Department of Physiology, Faculty of Medicine, University of Szeged, 6720 Szeged, Hungary; Department of Physiology, Anatomy and Neuroscience, Faculty of Sciences, University of Szeged, 6726 Szeged, Hungary; Department of Physiology, Faculty of Medicine, University of Szeged, 6720 Szeged, Hungary; Department of Physiology, Anatomy and Neuroscience, Faculty of Sciences, University of Szeged, 6726 Szeged, Hungary; Synthetic and Systems Biology Unit, Biological Research Centre, 6726 Szeged, Hungary; Department of Physiology, Faculty of Medicine, University of Szeged, 6720 Szeged, Hungary; Synthetic and Systems Biology Unit, Biological Research Centre, 6726 Szeged, Hungary; Institute for Molecular Medicine Finland (FIMM), University of Helsinki, 00014 Helsinki, Finland; Department of Physiology, Anatomy and Neuroscience, Faculty of Sciences, University of Szeged, 6726 Szeged, Hungary; Department of Physiology, Faculty of Medicine, University of Szeged, 6720 Szeged, Hungary; Neuroscience Division, Cardiff University, Cardiff CF10 3AX, UK

**Keywords:** brain state, interneuron, thalamocortical, thalamus

## Abstract

State-dependent thalamocortical activity is important for sensory coding, oscillations, and cognition. The lateral geniculate nucleus (LGN) relays visual information to the cortex, but the state-dependent spontaneous activity of LGN neurons in awake behaving animals remains controversial. Using a combination of pupillometry, extracellular, and intracellular recordings from identified LGN neurons in behaving mice, we show that thalamocortical (TC) neurons and interneurons are distinctly correlated to arousal forming two complementary coalitions. Intracellular recordings indicated that the membrane potential of LGN TC neurons was tightly correlated to fluctuations in pupil size. Inactivating the corticothalamic feedback to the LGN suppressed the arousal dependency of LGN neurons. Taken together, our results show that LGN neuronal membrane potential and action potential output are dynamically linked to arousal-dependent brain states in awake mice, and this might have important functional implications.

## Introduction

Brain states can fluctuate on various timescales and can profoundly influence neural and behavioral responses (McGinley, Vinck, et al. 2015b). This is most apparent when spontaneous neuronal activity and responses to sensory stimuli are compared between states of sleep and wakefulness (Livingstone and Hubel 1981; Massimini et al. 2005); but recently, the prominent influence of spontaneous variations within the waking state on both cortical neuronal responses and perceptual abilities has been documented in both humans (Fox and Raichle 2007) and rodents (Reimer et al. 2014; McGinley, David, et al. 2015a; Vinck et al. 2015). These state-dependent activities are reliant upon finely tuned interactions between neocortical and thalamic excitatory and inhibitory neuronal assemblies, strongly influenced by neuromodulatory inputs, and lead to various forms of rhythmic activities in thalamo-cortico-thalamic networks (Lőrincz and Adamantidis 2017). Driven by intrinsic and network mechanisms, state-dependent thalamic oscillations are thought to possess both a rhythm-regulation function and a plasticity function important for the faithful sensory information processing during attentive wakefulness (Crunelli et al. 2018).

Despite the large body of work aiming to identify the cellular and network mechanisms of thalamic low vigilance state oscillations, relatively little is known about the activity of thalamic neurons during brain state changes within the waking state. During relaxed wakefulness, lateral geniculate nucleus (LGN) thalamocortical (TC) neurons and interneurons in cats fire phase locked to and contribute to the generation of EEG α (8–13 Hz) rhythms characteristic of inattentive states (Hughes et al. 2004; Lőrincz et al. 2009). Both baseline activity and visual responses are markedly altered between attentive and inattentive states in LGN neurons of immobile rabbits (Bezdudnaya et al. 2006; Cano et al. 2006). Interestingly, mouse LGN neuronal activity was claimed not to be state dependent, as locomotion, a state associated with high arousal (Vinck et al. 2015) and an increase in the gain of visual cortical responses (Niell and Stryker 2010; Bennett et al. 2013; Polack et al. 2013; Reimer et al. 2014) failed to alter the spontaneous and visually evoked firing rate (FR) of LGN neurons (Niell and Stryker 2010). Despite the lack of classical α rhythms in rodents, some studies have revealed a pronounced 3–6 Hz oscillation in the mouse visual system, a potential transient analogue of α rhythms (Bennett et al. 2013; Einstein et al. 2017; Senzai et al. 2019) suggesting a strong state dependency of the visual thalamocortical system.

To resolve this controversy, we sought to test the arousal-dependent activity of single identified LGN neurons, revealing its origin and mechanisms involved. Using a combination of extra- and intracellular recordings from identified LGN neurons in awake behaving mice, pupillometry and pharmacological inactivation, we show that 1) spontaneous activity in thalamic neurons is correlated with state transitions in a cell type-specific manner, with TC neurons being positively and putative LGN interneurons negatively correlated to arousal; 2) the membrane potential of LGN neurons correlates with spontaneous fluctuations in pupil diameter providing a mechanistic explanation to the observed phenomenon, consistent with previous studies conducted in the neocortex (Reimer et al. 2014; McGinley, Vinck, et al. 2015b; Vinck et al. 2015); and 3) inactivating the corticothalamic feedback from V_1_ suppresses the state dependence of LGN activity, revealing its origin. These results can have important implications for the state-dependent function of thalamo-cortico-thalamic networks.

## Materials and Methods

All experimental procedures were performed according to the European Communities Council Directives of 1986 (86/609/EEC) and 2003 (2003/65/CE) for animal research and were approved by the Ethics Committee of the University of Szeged. Thirty-six C57BL/6 mice of either sex aged 2–6 months were used in this study.

### Surgical Preparation

Mice were anesthetized with ketamine/xylazine (100 and 10 mg/kg, respectively) and mounted in a stereotaxic frame (Model 902, David Kopf Instruments, USA), the skull of the animal exposed, and a stainless steel head post cemented over the frontal suture with dental acrylic resin (Pattern Resin LS, GC America, USA). Craniotomy positions were marked at the following stereotaxic coordinates: LGN: 2.3 mm posterior, 2.1 mm lateral to Bregma; primary visual cortex (V_1_): 3.3 mm posterior, 2.5 mm lateral to Bregma.

For postoperative care, mice received Rimadyl (5 mg/kg, intraperitoneally, Pfizer, USA) and an intramuscular injection of Gentamycin (0.1 mg/kg). Following an initial recovery from surgery of at least 5 days, mice were handled gently each day for 7 days to reduce excessive stress or anxiety during the recording sessions and the duration of the head fixation procedure gradually increased. On the day of the recording, small craniotomies (0.8–1 mm) were performed under isoflurane anesthesia (Forane, Abbvie, USA; dose: 1 L/min 1–1.5% isoflurane and 99–98.5% O_2_) at the positions previously marked leaving the dura mater intact. Mineral oil applied on the dural surface prevented dehydration. Mice were then transferred to an in vivo electrophysiological recording setup where their head posts were clamped in a custom apparatus and recording sessions started at least 60 min following awakening from anesthesia.

### Pupillometry

Pupillometry was conducted with a Genie (Teledyne Dalsa, Canada) high frame rate (300 fps) infrared camera focused on the eye of the animal illuminated with an infrared LED (900 nm, Marubeni Corp).

**Figure 1:**
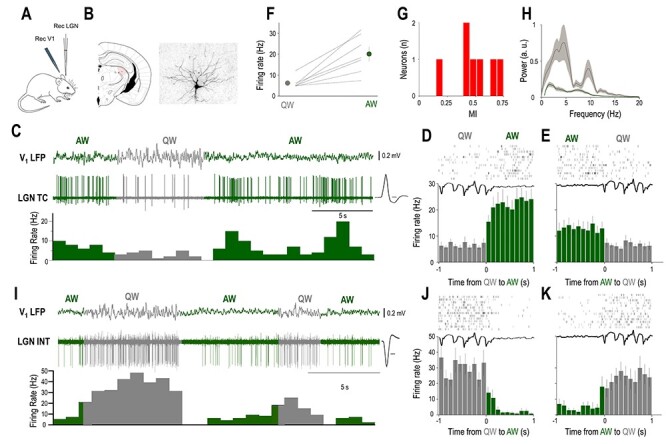
The baseline firing of identified LGN neurons is brain state dependent. (*A*) Schematics of the experimental setup. (*B*) Coronal brain section shows the location and morphology of a neuron recorded and labeled in the LGN. (*C*) Example of simultaneous V_1_ LFP and unit recording from the LGN TC neuron shown in *B* in an awake head-restrained mouse. Spontaneous state transitions are color coded for clarity (AW—green; QW—gray). Averaged action potential waveform is shown on the right, calibration: 0.2 ms. (*D*, *E*) Schematic state transition LFP (top), raster plot of the LGN TC neuron (middle), and peri-event histogram (bottom); state transitions color coded as in (*C*). (*F*) Mean FR changes between QW and AW for all identified LGN TC neurons (*n* = 7). (*G*) Distribution of MIs for all identified LGN TC neurons (*n* = 7). (*H*) Power spectrum of the QW and AW states for all the recordings from morphologically identified TC neurons (*n* = 7). (*I*) Example of simultaneous V_1_ LFP and unit recording from a putative LGN interneuron in an awake head-restrained mouse. Note the high baseline FR during QW. Averaged action potential waveform is shown on the right, calibration: 0.2 ms. (*J*, *K*) Schematic LFP state transition (top), raster plot of the putative LGN interneuron (middle), and peri-event histogram (bottom); state transitions color coded as in (*B* and *I*).

### In Vivo Electrophysiology and Juxtacellular Labeling

Single-unit extracellular and local field potential (LFP) recordings from the LGN and V_1_ were performed using either glass micropipettes filled with 0.5 M NaCl solution containing 1.5% w/v Biocytin (Sigma Aldrich, USA, impedance 3–25 MΩ) or Silicon probes (single shank, 32-channel, Neuronexus). Intracellular recordings, using the current-clamp technique, were performed with standard wall glass microelectrodes filled with 1 M potassium acetate (impedance 30–50 MΩ). When advancing the electrode through the LGN, the voltage output (*V*_out_) of the amplifier has regularly (every minute) been zeroed and the bridge continuously balanced throughout the recordings. At the end of the impalement, the *V*_out_ was read and the recorded *V*_m_ adjusted if necessary. Recorded neurons were included in the data set only if their resting membrane potential during the active period was more hyperpolarized than −45 mV and had overshooting action potentials.

The biological signals were pre-amplified with Axon HS-9A headstages (Molecular Devices) and amplified with an Axoclamp 900A amplifier (Molecular Devices) and filtered (0.1 Hz−200 Hz for LFP, 0.3–6 kHz for units). The amplified signals were then digitized with a CED Power3 1401 AD converter (Cambridge Electronic Design) at 30 kHz sampling rate using Spike2 software (Cambridge Electronic Design). Signals recorded with Silicon probes were amplified, filtered, and digitized using an Intan circuit board (RHD2000, Intan Technologies).

To confirm the morphology and location of the recorded cells as LGN neurons, we performed juxtacellular labeling of LGN cells (*n* = 7) by applying pulses of anodal current (1–4 nA, 500 ms, 50% duty cycle) for 2–5 min (Pinault 1996). At the end of a recording session (maximum one neuron was labeled per animal), overanesthetized mice were transcardially perfused with cold phosphate-buffered solution (PB, 100 mM, pH: 7.4), followed by 4% paraformaldehyde (PFA), the brain removed and transferred to 4% PFA solution in 100 mM PB and stored at 4°C overnight. 50-μm coronal brain sections containing the LGN were sliced with a vibratome (VT1000S, Leica, Germany). During the histological processing, slices were cryoprotected with 10% and 20% sucrose solution, antibody penetration increased by using a freeze–thaw method (Somogyi and Takagi 1982). After 2 h of incubation with Cy3-Streptavidin (Jackson ImmunoResearch), slices were mounted on glass slides and covered with cover slips. A BX60 fluorescent microscope (Olympus, Japan) and a Surveyor software (Objective Imaging, UK) were used to visualize the labeled neurons.

### Cortical Inactivation with Muscimol

For inactivation of the V_1_, we microinjected muscimol, a GABA_A_ receptor agonist (200 nL, 1 mM), through a glass pipette (15 μm tip diameter) at coordinates 3.3 mm posterior; 2.3 mm lateral to Bregma at a depth of 0.5 mm from brain surface. The correlation of the LGN neuron baseline activity and pupil diameter was quantified and compared with control (*n* = 7 neurons). Saline injections (200 nl) into the V_1_ did not affect the correlation of LGN neuron baseline activity and pupil diameter (*n* = 5 neurons).

**Figure 2:**
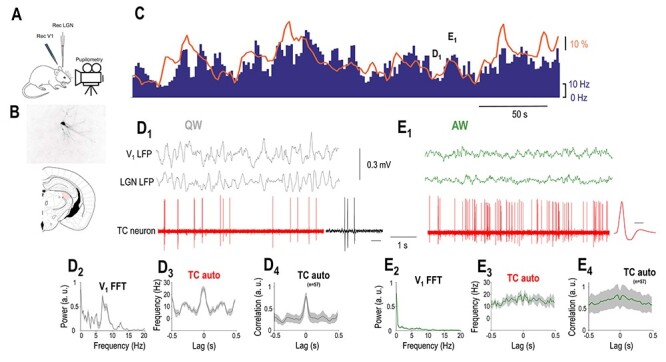
LGN TC neurons are positively correlated to arousal. (*A*) Schematics of the experimental setup. (*B*) Coronal brain section shows the location and morphology of a TC neuron recorded and labeled in the LGN. (*C*) FR of the LGN TC neuron shown in *B* (blue bars) increases when the simultaneously recorded pupil (orange line) is dilated but decreases when the pupil is constricted. (D_1_) Simultaneously recorded V_1_ LFP, thalamic LFP, and single units of the TC neuron shown in *B* and *C* shown on a faster time-base during a period of constricted pupil (marked in *C*) corresponding to a state of QW. Note the presence of putative LTS-mediated bursts enlarged on the right (calibration: 50 ms). (*D*_2_) V_1_ power spectrum, (*D*_3_) autocorrelation of the TC neuron shown in *B* and *C*, and (*D*_4_) grand average autocorrelation of all LGN TC neurons during periods of constricted pupil. (*E*_1_) Simultaneously recorded V_1_, thalamic LFP, and single units of the TC neuron shown in *B* and *C* plotted on a faster time-base during a period of dilated pupil (marked on *C*) corresponding to a state of AW. The average action potential waveform is shown on the right (time calibration: 0.5 ms). (*E*_2_) V_1_ power spectrum, (*E*_3_) autocorrelation of the TC neuron shown in *B* and *C*, and (*E*_4_) autocorrelation of all putative LGN TC neurons during periods of dilated pupil.

### Data Analysis

Periods of NREM and REM sleep identified by cortical LFP analysis and periods of rhythmic eye movements were excluded from data analysis. Data analysis was performed off-line with custom-written MATLAB and ImageJ routines.

Pupillometry was carried out offline on recoded AVI files using a custom ImageJ plug-in. The plug-in extends the Snakuscules model (Thevenaz and Unser 2008) for video files. The model is designed to find circular objects using a variational framework, by minimizing an energy function. The model consists of a circle and a ring on it such that their area is always the same. In other words, the radii of the inner and outer circle always have the same ratio. The energy function is simply the difference of the intensities covered by the ring and the circle, which is minimal when the circle fits a circular (bright) object and the ring is in the background (dark). Initially, a rectangular region in the iris is manually selected for reference pixel intensities, which are then used to invert the image such that this region is dark and the pupil white. Afterwards, the user has to provide the segmentation manually in the first frame of the video. Assuming that the change between two consecutive frames is small (due to the 50 fps camera used), the software uses the segmentation of the previous frame as an initial solution for the subsequent frame. The results are then written to a CSV file, including the frame index, pupil position, and radius. Black frames that are used for synchronization of the video files and electrophysiology are also detected and indicated in the result file. Segmentation is not performed in black frames. For comparing the FRs of thalamic neurons with respect to the pupil diameter, the upper and lower terciles of the pupil diameter distributions were used.

LGN neurons were identified as TC or interneurons based on either their morphology (*n* = 7 TC neurons labeled), action potential duration (<0.3 ms for LGN interneurons, >0.3 ms for TC neurons, see Fig. 4*A*), and action potential height ratio (Fig. 4*A*). A burst in thalamic neurons was defined as a cluster of spikes consisting of minimum of two action potentials, a maximum interspike interval of 10 ms, and had to be separated from other bursts by more than 100 ms.

Brain states were detected from the V_1_ LFP signal using a semi-automated level threshold method in Spike2 (CED, UK). Quiet wakefulness (QW) states were defined as periods of at least 3 s with large amplitude (2× baseline) 3–6 Hz voltage fluctuations. The timing of state changes was determined by detecting the peak of the first 3–6 Hz oscillation cycle for active wakefulness (AW) to QW transitions and of the last peak of the 3–6 Hz oscillation cycle for QW to AW transitions. Periods of dilated pupil correspond to the upper tercile and periods of constricted pupil to the lower tercile of the pupil distribution for a given recording.

For correlating pupil diameter and FRs, we used the random permutation test, in which two data points were randomly selected and paired up from the FR and pupil data points (i.e., one from each), and this was repeated 1000 times, resulting in a (normal) distribution of random data pairs serving as null hypothesis, and two-tailed *P*-values were calculated. Pearson’s *r* calculated from the real (observed) FR/pupil diameter data was then compared to the random distribution, and *r* values falling out of 95% confidence intervals were considered statistically significant.

The modulation index (MI) was calculated using the following formula: FR_AW_ − FR_QW_/FR_AW_ + FR_QW_. Hence an MI value of 1 corresponds to the strongest possible excitation and a value of −1 corresponds to the strongest possible suppression during a QW to AW state change.

## Results

We performed extracellular and intracellular recordings of identified thalamic neurons with simultaneous LFP in the primary visual cortex (V_1_ LFP) and pupillometry in awake head-restrained mice to reveal the brain state-dependent activity of thalamic neurons in the LGN.

**Figure 3:**
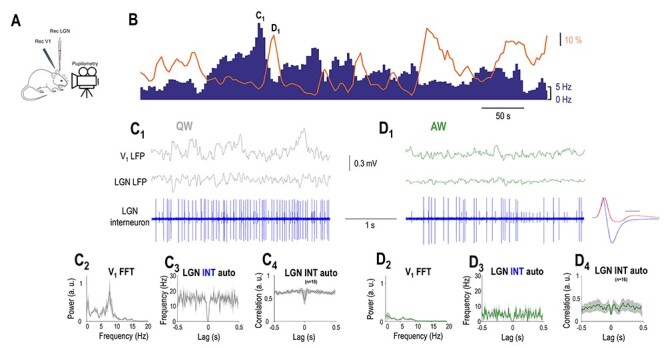
LGN interneurons are negatively correlated to arousal. (*A*) Schematics of the experimental setup. (*B*) FR of an example LGN interneuron (blue bars) decreases when the simultaneously recorded pupil (orange line) is dilated but increases when the pupil is constricted. (*C*_1_) Simultaneously recorded visual cortical, thalamic LFP, and single units of the LGN interneuron shown in *B* plotted on a faster time-base during a period of constricted pupil (marked in *B*) corresponding to a state of QW. (*C*_2_) V_1_ power spectrum, (*C*_3_) autocorrelation of the putative LGN interneuron shown in *B* during 10 consecutive periods of constricted pupil, and (*C*_4_) grand average normalized autocorrelation of all recorded LGN interneurons during periods of constricted pupil. (*D*_1_) Simultaneously recorded V_1_, thalamic LFP, and single units of the putative LGN interneuron shown in *B* plotted on a faster time-base during a period of constricted pupil (marked in *C*) corresponding to a state of AW. The average action potential waveform is shown in the right (blue, time calibration: 0.5 ms) with the average action potential of the TC neuron (red) from Figure 2 overlaid for comparison. Note the presence of a second neuron (smaller spikes) not correlated with the pupil diameter. (*D*_2_) V_1_ power spectrum, (*D*_3_) autocorrelation of the LGN interneuron shown in *B* during 10 consecutive periods of dilated pupil, and (D_4_) grand average normalized autocorrelation of all recorded LGN interneurons during periods of dilated pupil.

**Figure 4:**
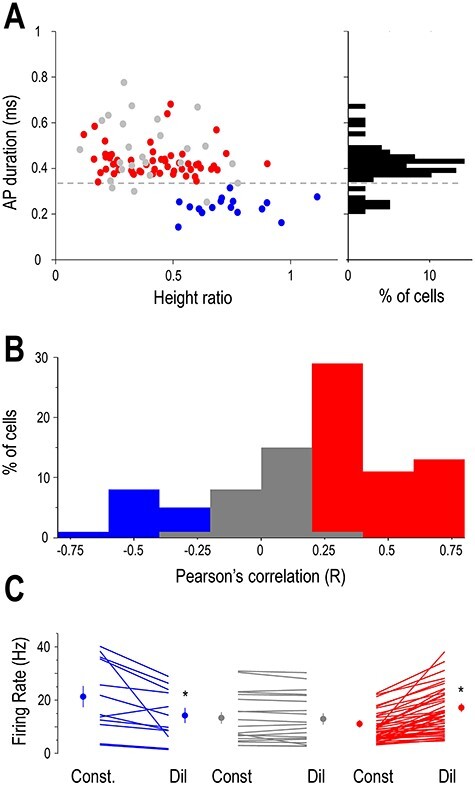
Arousal-dependent activity in the LGN depends on neuronal identity. (*A*) Classification of neurons based on spike waveform parameters. Scatter plot of duration versus height ratios for recorded LGN units (red: neurons positively correlated with pupil diameter, gray: uncorrelated neurons, blue: neurons negatively correlated with pupil diameter). The histogram of the action potential durations is shown on the right. Putative LGN interneurons are seen for durations ˂0.35 ms and putative TC neurons for durations >0.35 ms. (*B*) Distribution of pupil diameter/FR correlations. Putative LGN interneurons were negatively correlated (left, blue bars) and TC neurons were either uncorrelated (middle, gray bars) or positively correlated (right, red bars). (*C*) Mean FRs of individual neurons during periods of constricted (Const) and dilated (Dil) pupil. Group averages are shown on the side of each group for the two states, color codes same as in *A*.

### The Baseline Activity of Most LGN Neurons Correlates with Brain States

States of QW were characterized by large-amplitude slow V_1_ LFP fluctuations (mean peak to peak amplitude 0.77 ± 0.2 mV, *n* = 98) and periods of active wakefulness (AW) by small-amplitude fast fluctuations (mean peak to peak amplitude 0.27 ± 0.11 mV, *n* = 98) in the LFP recorded in V_1_ (Fig. 1*C*). State transitions were accompanied by prominent changes in the activity of all morphologically identified LGN TC neurons (Fig. 1*B*,*C*, *n* = 7). QW to AW transitions led to an increase in firing (Fig. 1*D*), but AW to QW transitions led to a decrease in firing (Fig. 1*E*). The baseline activity of all morphologically identified LGN TC neurons (*n* = 7) decreased their spontaneous firing during QW compared to AW (QW: 6.2 ± 0.99, AW: 20.1 ± 3.4; *P* < 0.001, Wilcoxon’s signed-rank test, Fig. 1*F*), but the strength of this modulation varied between neurons as shown on the distribution of their MIs (Fig. 1*G*). A subset of LGN neurons characterized by high baseline FR (Fig. 1*I*) also showed state transition-related changes of baseline activity. In this neuronal subset, QW to AW transitions led to a decrease in firing (Fig. 1*J*), but AW to QW transitions led to an increase in firing (Fig. 1*K*).

To further explore the relationship between arousal and thalamic neuronal firing within the waking state, we recorded the spontaneous firing of LGN neurons with simultaneous V_1_ and thalamic LFP and pupillometry. The baseline activity of most recorded thalamic neurons (73/98, 74%) correlated with the pupil diameter (*P* < 0.05, random permutation test). The baseline activity of the remaining neurons (*n* = 25/98, 26%) did not correlate with the pupil diameter (*P* > 0.05 for the non significant, random permutation test). In the majority of the state-modulated neurons, FRs showed a statistically significant positive correlation with pupil diameter (57 of 73 significant cells *P* < 0.05, random permutation test; mean Pearson’s *r* = 0.411 ± 0.025, Fig. 2*A*,*B*). Both neurons positively correlated with pupil diameter and nonmodulated neurons were classified as TC neurons either morphologically (*n* = 7) or using physiological criteria, as follows. The duration of the action potentials was relatively large (positively modulated: 0.43 ± 0.01 ms, nonmodulated: 0.48 ± 0.02 ms) and homogeneous (the action potential durations were not significantly different in the two groups, *P* > 0.05, Wilcoxon rank-sum test) in both groups. Figure 2*B* illustrates an example morphologically identified LGN TC neuron showing a positive correlation with pupil diameter. Note that the periods of constricted pupil coincide with QW states and putative low-threshold spike (LTS) mediated burst firing of the LGN TC neuron (Fig. 2*D*_1_), whereas the periods of dilated pupil with AW states and high-frequency tonic action potential output (Fig. 2*D*_2_).

Thus, periods of pupil constriction correspond to QW states as the V_1_ LFP shows a clear peak in the 3–6 Hz band (Figs 2*D*_2_ and 3*C*_2_) and the autocorrelation of the TC neuron low-frequency rhythmic burst firing (Fig. 2*D*_3_,*D*_4_). On the other hand, periods of pupil dilation correspond to AW states as the V_1_ LFP shows no peak in the 3–6 Hz band (Fig. 2*E*_2_) and the autocorrelation of the TC neuron reveals high-frequency tonic firing (Fig. 2*E*_3_,*E*_4_). Both the FR changes (Fig. 4*C*) and the firing mode changes (Figs 2*D*_4_,*E*_4_ and 4*C*) of putative LGN TC neurons held true at the population level.

Strikingly, we found a smaller percentage of neurons showing negative correlation to pupil diameter (16/98, 16%, *P* < 0.05, random permutation test; mean Pearson’s *r* = −0.4 ± 0.045). The duration of the action potentials in these neurons was significantly narrower than in TC neurons (0.23 ± 0.01 ms in negatively correlated neurons vs. 0.44 ± 0.01 in putative TC neurons, *P* < 0.05, Wilcoxon rank-sum test), and the waveform of their action potential was more biphasic (height ratio: 0.41 ± 0.02 in putative TC neurons, 0.73 ± 0.04 in putative LGN interneurons, *P* < 0.05, Wilcoxon rank-sum test) (Figs 3*D*1 and4A). These neurons were classified as putative LGN interneurons. Figure 3*B* illustrates an example putative LGN interneuron showing a negative correlation with pupil diameter. Note that during the periods of constricted pupil characteristic of QW states (Fig. 3*C*_1_,*C*_2_) this putative LGN interneuron is more active than during AW (compare Fig. 3*C*_1_,*C*_3_,*D*_1_,*D*_3_). This was the case for the whole population of putative LGN interneurons (Fig. 4*C*). Also note that the activity of the putative LGN interneuron consists of tonic action potential output during both QW (Fig. 3*C*_1_,*C*_3_) and AW (Fig. 3*D*_1_,*D*_3_). This was the case for the whole population of putative LGN interneurons (Fig. 3*C*_4_,*D*_4_). Thus, LGN neurons in awake behaving mice form three groups where the spontaneous firing is subject to arousal-dependent modulation being negatively correlated with the pupil diameter in putative LGN interneurons and positively in most putative TC neurons while a subset of putative TC neurons does not show a significant correlation (Fig. 4*B*).

### The Membrane Potential of LGN TC Neurons Is Correlated with Brain States

**Figure 5:**
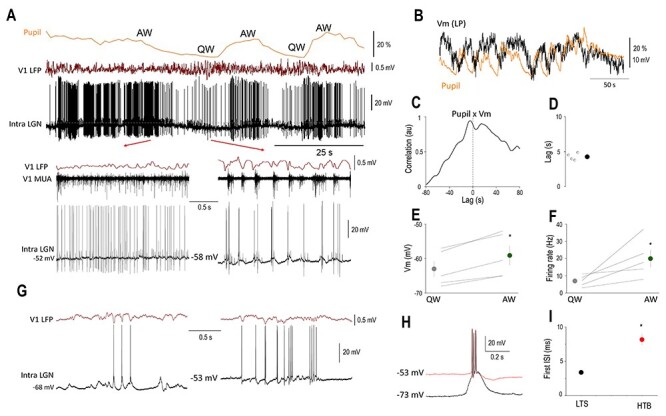
The membrane potential of LGN TC neurons is correlated with brain states. (*A*) Simultaneous pupillometry, cortical LFP, MUA, and LGN *V*_m_ recording (dotted line indicates −50 mV) during state transitions in an awake head-restrained mouse. Quiet wake (QW) and active wake (AW) states are indicated above the pupillometry trace and shown on a faster time-base below. Note the large retinogeniculate EPSPs on the *V*_m_ recording. (*B*) Slow rhythmic fluctuations in the pupil diameter are correlated with the *V*_m_ of this LGN neuron as apparent on the low-pass filtered *V*_m_ (*V*_m_ LP) overlaid on the pupillometry trace. (*C*) Normalized cross-correlation and (*D*) quantification of *V*_m_ delay in respect to pupil diameter. Mean FRs (*E*) and *V*_m_ (*F*) for the two brain states. (G) LTS and HT bursting are present in the same LGN TC neuron during QW states. During periods of relatively hyperpolarized membrane potentials (left), LTS-mediated bursts accompany V_1_ LFP 3–6 Hz oscillations, but HT bursts are present at less hyperpolarized membrane potentials (right). (*H*) Ovarlaid LTS (black trace) and HT bursts (red trace) recorded from the neuron in (*G*). (*I*) Mean duration of the first ISI from LTS and HT bursts, color codes as in (*H*).

To reveal the mechanisms underlying the state-dependent fluctuation of thalamic neurons, we performed intracellular recordings of LGN thalamocortical neurons (*n* = 5) of awake mice while simultaneously monitoring the pupil diameter (*n* = 4) and recorded the LFP and multi-unit activity (MUA) in V_1_ (Fig. 5*A*). In all the neurons recorded, we found a clear-cut correlation between the membrane potential and pupil diameter (Fig. 5*B*), such that periods of pupil constriction were associated with low baseline FRs (6.98 ± 3.19), hyperpolarized membrane potentials (−63.0 ± 2.3 mV), and burst firing in two of the neurons recorded (Fig. 5*A* bottom right, 5G). Importantly, these bursts were recorded at a membrane potential inconsistent with LTS burst (Fig. 5*H*) (Lőrincz et al. 2009; Crunelli et al. 2012, 2018). In addition, the interspike interval of the first and second action potentials in LTS and HT bursts was different (LTS: 3.38 ± 0.15 ms, HTB: 8.15 ± 2.69 ms, *P* < 0.001, Wilcoxon rank-sum test, Fig. 5*I*) and is therefore thought to represent high threshold bursts (Hughes et al. 2004; Lőrincz et al. 2008, 2009; Crunelli et al. 2012). Periods of pupil dilation, on the other hand, were associated with high frequency (19.96 ± 10.98 Hz, Fig. 5*A*,*F*) tonic action potential output and less hyperpolarized membrane potentials (−59.1 ± 2.82 mV, Fig. 5*A*,*E*). When quantifying the correlation of the low-pass filtered membrane potential and pupil diameter, we found that the membrane potential of LGN TC neurons was lagging the changes in pupil diameter by approximately 5 s (Fig. 5*B*–*D*). Large amplitude retinogeniculate EPSPs were not state dependent (mean EPSP rate QW: 17.75 ± 3.88 Hz, AW: 19.35 ± 4.36 Hz, *P* > 0.05, Wilcoxon’s signed-rank test). Thus, the state-dependent action potential output of thalamic neurons can be accounted for by slow changes in their neuronal membrane potential.

### Inactivation of the Visual Cortex Suppresses the Brain State Correlation of LGN Neurons

**Figure 6:**
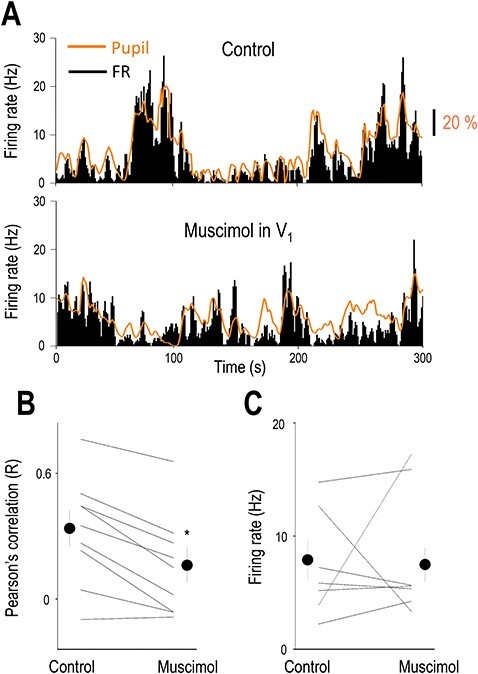
V_1_ inactivation decreases the correlation of LGN neuron firing and pupil diameter. (*A*) Baseline activity of an example thalamic neuron and the simultaneously recorded pupil diameter before (control) and following V_1_ inactivation. Note the spontaneous fluctuations in pupil diameter and thalamic firing in both conditions. (*B*) Correlation between the pupil diameter and thalamic baseline activity before (Control) and following muscimol infusion in V_1_. (*C*) Firing rate of thalamic neurons before (Control) and following muscimol infusion in V_1_.

As rhythmic synchronous LGN-driven EEG activities are influenced by cortical feedback to the thalamus (Lőrincz et al. 2009) we monitored the effects of inactivating the cortical feedback from V_1_ to the LGN and its effect on the arousal-dependent activity of LGN neurons. Figure 6*A* shows the baseline activity of an example thalamic neuron and the simultaneously recorded pupil diameter. In the control condition (Fig. 6*A* top), the FR and pupil diameter changes are relatively simultaneous but following V_1_ inactivation periods of dilated pupil are not always followed by an increase in FR and FR changes do not always coincide with a change in pupil diameter. To quantify the relationship between pupil diameter and thalamic baseline firing in the two conditions, we compared the FR of thalamic neurons and calculated the correlation between FR and pupil diameter before and following V_1_ inactivation. The FR of individual neurons before and after V_1_ inactivation varied considerably (Fig. 6*B*) but did not reach statistical significance as a group (control: 7.93 ± 1.69 Hz, V_1_ inactivation: 7.52 ± 1.47 Hz, *P* > 0.05, Wilcoxon’s signed-rank test, *n* = 9). When comparing the correlation of thalamic single units and pupil diameter before and following V_1_ inactivation (Fig. 6*C*), we found a significant decrease (control: 0.28 ± 0.07, V_1_ inactivation: 0.13 ± 0.06, *P* < 0.05, Wilcoxon’s signed-rank test, *n* = 9), suggesting that corticothalamic input from V_1_ is at least partly responsible for the state-dependent activity in LGN TC neurons.

## Discussion

Thalamocortical dynamics are essential for sensory coding, cognition, and brain rhythms. Whereas cortical function in general and visual cortical activity in particular are well known to be modulated by the level of arousal in awake animals (McGinley, Vinck, et al. 2015b), the arousal-dependent activity of reciprocally connected visual thalamus is more controversial. Here, in awake behaving mice, we found that 1) the baseline LGN neuronal activity correlates with arousal during the awake state; 2) the polarity of this correlation is cell type specific, with LGN TC neurons being positively, and putative LGN interneurons negatively correlated with arousal; and 3) this state-dependent activity at least partly originates from cortical feedback.

LGN activity has long been known to differ during wakefulness compared to sleep (Hirsch et al. 1983), during alert and inattentive states (Bezdudnaya et al. 2006), with a subset of bursting LGN TC neurons playing an important role in generating, while putative LGN interneurons in providing tonic firing TC neurons with temporally precise phasic inhibition during individual cycles of the alpha rhythm (Lőrincz et al. 2009). Some results suggested that LGN spontaneous and visually evoked FRs are not different between immobile and running mice, whereas V_1_ activity was markedly different between the two states (Niell and Stryker 2010). This controversy might arise from differences in the definition of brain states: whereas locomotion is only associated with alert wakefulness, both quiet and alert wakefulness can occur during immobility (Vinck et al. 2015). Pupil diameter is an excellent proxy for a general neuromodulatory tone and brain states (Reimer et al. 2016), and its combination with V_1_ LFP level of synchronization as used in the present study provides a more accurate state definition.

Our results reveal an arousal-dependent spontaneous activity in the majority of LGN neurons of awake behaving animals. A recent study found that TC neurons of the primary and higher order somatosensory thalamus also show state-dependent activity when comparing activities recorded during QW and sleep (Urbain et al. 2019). Notably, the polarity of the correlation between neuronal activity and arousal, quantified by monitoring the pupil diameter, depends on neuronal identity within the LGN. Specifically, whereas TC neurons, the dominant cell type in the LGN, tend to increase their activity during AW (desynchronized V_1_ LFP and dilated pupil), putative LGN interneurons, which are key for intimately controlling the timing of TC neuron firing in awake animals (Lőrincz et al. 2009), behave in an opposite manner showing decreased activity during AW and increased activity during QW (synchronized V_1_ LFP and constricted pupil). Taken together, the increase in firing in most TC neurons during AW is accompanied by a decrease in inhibition derived from local interneurons (Acuna-Goycolea et al. 2008; Lőrincz et al. 2009). This may have implications for both the spontaneous activity of LGN TC neurons and their sensory coding. The decrease in firing in putative LGN interneurons during AW does not necessarily mean that the net inhibition in TC neurons is decreased as some thalamic reticular neurons, the sources of a much larger inhibitory conductance (Bal et al. 1995), are known to fire at much higher rates during AW (Gardner et al. 2013; Halassa et al. 2014). The significance of this arousal-dependent modulation of inhibition derived from local interneurons in rhythmic brain activity and sensory coding needs to be addressed by future studies.

What mechanisms are responsible for the differential arousal-dependent activity of LGN TC and interneurons? Our intracellular recordings in TC neurons reveal that during AW states the membrane potential of LGN TC neurons is less hyperpolarized than during QW, providing an explanation for the increases in FR during AW in LGN TC neurons. The firing mode of these neurons also changed from tonic to burst firing in the QW state, and in some cases, these bursts were characterized by a membrane potential polarization and interspike interval inconsistent with LTS-mediated burst firing, suggesting that they are high-threshold bursts similar to the ones recorded in the LGN of behaving cats (Hughes et al. 2004; Lőrincz et al. 2009) and the somatosensory thalamus of lightly anesthetized mice (Crunelli et al. 2012). Some of our extracellular recordings during QW clearly show LTS-mediated bursts (Fig. 2*D*_1_), suggesting that high-threshold bursting is a property of a subset of LGN TC neurons as it was described in the cat LGN (Hughes et al. 2004; Lőrincz et al. 2008, 2009). Importantly, we revealed a tight correlation between TC neuron membrane potential and pupil diameter, suggesting that the arousal-dependent membrane potential might originate from differential neuromodulation during AW and QW states. Multiple brain sources could contribute to this modulation, the most prominent being corticothalamic fibers (Wilson et al. 1984). Indeed, upon V_1_ inactivation, we found a prominent suppression of the arousal dependency of LGN TC neurons, suggesting that corticothalamic feedback is at least partly responsible for this phenomenon. Taken together, our results show that the membrane potential and action potential output of LGN neurons are dynamically linked to arousal-dependent brain states in awake mice, and this fact might have important functional implications.

## Funding

Hungarian Scientific Research Fund (grants NN125601 and FK123831 to M.L.L.); the Hungarian Brain Research Program (grant KTIA_NAP_13-2-2014-0014 to M.L.L.); Ministry of Human Capacities, Hungary (20391-3/2018/FEKUSTRAT).

## Notes

M.L.L. is a grantee of the János Bolyai Fellowship. *Conflict of Interest*: None declared.
